# Tailored Lipoprotein‐Like miRNA Delivery Nanostructure Suppresses Glioma Stemness and Drug Resistance through Receptor‐Stimulated Macropinocytosis

**DOI:** 10.1002/advs.201903290

**Published:** 2020-01-20

**Authors:** Gan Jiang, Huan Chen, Jialin Huang, Qingxiang Song, Yaoxing Chen, Xiao Gu, Zhenhuan Jiang, Yukun Huang, Yingying Lin, Junfeng Feng, Jiyao Jiang, Yinghui Bao, Gang Zheng, Jun Chen, Hongzhuan Chen, Xiaoling Gao

**Affiliations:** ^1^ Department of Pharmacology and Chemical Biology Shanghai Universities Collaborative Innovation Center for Translational Medicine Shanghai Jiao Tong University School of Medicine 280 South Chongqing Road Shanghai 200025 China; ^2^ Department of Neurological Surgery Renji Hospital Shanghai Jiao Tong University School of Medicine 1630 Dongfang Road Shanghai 200127 China; ^3^ Key Laboratory of Smart Drug Delivery Ministry of Education School of Pharmacy Fudan University 826 Zhangheng Road Shanghai 201203 China; ^4^ Department of Medical Biophysics and Ontario Cancer Institute University of Toronto Ontario M5G 1L7 Canada; ^5^ Institute of Interdisciplinary Integrative Biomedical Research Shuguang Hospital Shanghai University of Traditional Chinese Medicine 1200 Cailun Road Shanghai 201210 China

**Keywords:** glioma initiating cells, macropinocytosis, miRNA delivery, tailored lipoprotein‐like nanostructures

## Abstract

Glioma initiating cells (GICs) function as the seed for the propagation and relapse of glioma. Designing a smart and efficient strategy to target the GICs and to suppress the multiple signaling pathways associated with stemness and chemoresistance is essential to achieving a cancer cure. Inspired by the metabolic difference in endocytosis between GICs, differentiated glioma cells, and normal cells, a tailored lipoprotein‐like nanostructure is developed to amplify their internalization into GICs through receptor‐stimulated macropinocytosis. As CXCR4 is highly expressed on GICs and glioma tumor sites, meanwhile, the activation of CXCR4 induces the receptor‐stimulated macropinocytosis pathway in GICs, this CXCR4 receptor‐stimulated lipoprotein‐like nanoparticle (SLNP) achieves efficient accumulation in GICs in vitro and in vivo. By carrying microRNA‐34a in the core, this tailored SLNP reduces sex‐determining region Y‐box 2 and Notch1 expression, powerfully inhibits GICs stemness and chemoresistance, and significantly prolongs the survival of GICs‐bearing mice. Taken together, a tailored lipoprotein‐based nanostructure realizes efficient GICs accumulation and therapeutic effect through receptor‐stimulated macropinocytosis, providing a powerful nanoplatform for RNA interference drugs to combat glioma.

## Graphical abstract

A tailored lipoprotein‐based nanostructure is synthesized to realize glioma initiating cells (GICs)‐targeted accumulation through a receptor‐stimulated macropinocytosis manner. By carrying RNA interference drug in the core, this tailored nanostructure reduces sex‐determining region Y‐box 2 and Notch1 expression, powerfully inhibits GICs stemness and chemoresistance, and significantly prolongs the survival of GICs‐bearing mice, providing a powerful precision nanoplatform to combat glioma.

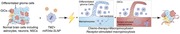

## 1 Introduction

Most cancers contain a tumorigenic subpopulation, known as tumor‐initiating cells (TICs), which is functionally defined by their self‐renewal and differentiation ability. In glioma, glioma initiating cells (GICs) are resistant to conventional chemotherapy, thereby leading to the propagation of highly invasive and metastatic cancers with only 9.8% 5‐year survival rate.^1^ Targeting GICs is thus critical to achieving glioma cure. However, due to the spatial and functional heterogeneity of GICs, the existing therapies including both chemotherapy and target therapy toward the specific signaling pathways or tumor microenvironments could not completely eliminate or inhibit GICs, thus resulting in poor prognosis.^2^ To combat glioma in a more efficient way, developing novel GICs‐targeting strategy which can efficiently target the heterogeneous GICs and suppress the multiple signaling pathways associated with GICs stemness and drug resistance is of great importance.

Cancer‐specific metabolism is a hallmark with therapeutic potential because cancer cells require altered metabolic states to realize proliferation and adaptation to stress.^3^ Indeed, the raised acquisition of nutrition is essential in tumorigenesis, and the so‐called “metabostemness” acts as a hallmark to reprogram normal cells or differentiated cancer cells into TICs.^4^ A critical metabolic adaptation in glioma and glioma initiating cells is macropinocytosis, an evolutionarily conserved clathrin‐independent endocytic pathway driven by actin. It was shown that macropinocytic uptake of extracellular proteins provided the needed amino acids for rapid‐growth cancer cells.^5^ Macropinocytosis can also induce GICs to internalize protein‐based drugs and exosomes.^6^ Given the fact that macropinocytosis is crucial for glioma cells and GICs to drink the extracellular nutrition, we proposed that strategies targeting and amplifying this metabolism diversity in GICs could be utilized for highly efficient GICs‐targeting drug delivery.

Macropinocytosis can be induced by growth factors, chemokines, cytokines, and pathogens.^7^ When the major tumorigenesis signaling pathway such as mutant KRas is activated, macropinocytosis emerges in a receptor‐independent mode.^8^ Thus, stimulation of macropinocytosis related‐receptors like epidermal growth factor receptor could simultaneously enhance the macropinocytic cellular uptake of extracellular nutrition.[qv: 6b] It has been found that more than 23 different types of cancers showed overexpression of CXCR4.^9^ More importantly, data derived from both clinical samples and glioma cell lines showed that CXCR4 expression was closely linked to CD133^+^ glioma stem cells.^10^ Especially, it was found that the 16 genes overexpressed above fivefold in glioma stem cells were also highly expressed in normal human adult brain, expect CXCR4. The increased expression of CXCR4 contributes to the survival, self‐renewal, and distant metastases of various GICs.^11^ Besides, as a coreceptor for extracellular amino acids, oligoarginine cell‐penetrating peptides, and other protein‐based vesicles, the activation of CXCR4 induces the highly efficient macropinocytosis pathway to facilitate the entrance of the nutrients into the cells.^12^ Therefore, CXCR4 could be a potent target for TICs‐targeting drug delivery through the CXCR4‐stimulated enhanced macropinocytosis.

Stromal cell‐derived factor 1 (SDF1, CXCL12) could bind to the CXCR4 receptor and thus stimulate the macropinocytosis pathway.^13^ Actually, the interaction between SDF1 and CXCR4 plays an essential role in the generation and maintenance of the perivascular stem cell niche, which is also involved in the chemoresistance and infiltration of GICs.^14^ CXCR4 possesses different binding pocket that can recognize multiple peptides, small molecules, and proteins, in which subtle structural changes in the ligands may lead to distinct interactions with CXCR4 and result in a full to partial function of SDF1.^15^ Through a structure‐based study, it is known that the CXCR4‐binding domain of SDF1 is RFFESH, while the activation domain is KPVSLSYR.^16^ To realize efficient GICs‐targeting drug delivery, it is essential to mimic the structure of SDF1, in order to not only selectively bind to the CXCR4 receptor but also mainly stimulate its downstream macropinocytosis signaling pathway. To justify this hypothesis, here we designed three SDF1 mimic peptides (RFFESH, KPVSLSYR, and RFFESHAPAKPVSLSYR), which is responsible for the binding, activation, and binding plus activation of CXCR4, to evaluate the potency of CXCR4‐stimulated macropinocytosis for GICs‐targeting drug delivery.

The inhibition of multiple signaling pathways associated with GICs stemness and drug resistance is another obstacle to cancer therapy, which may be treatable with RNA interference therapy. MicroRNAs (miRNAs), 21‐nt to 25‐nt‐long nucleotide fragments that induce the multiple target mRNAs degradation via association by a 7‐nt nucleotide seed sequence with a complementary sequence of the target mRNA, were taken as promising candidates.^17^ It has been demonstrated that miRNAs could control the self‐renewal and differentiation of normal stem cells, which are also linked with tumorigenesis. Thus, miRNAs could regulate the fate of GICs as well. Compared with differentiated cancer cells and normal cells, the miRNA expression profile of GICs showed significant changes, which were related to the self‐renewal, migration, invasion, and drug resistance of GICs.^18^ The crucial roles of miRNA in GICs may render the development of gene drugs to eliminate GICs. As a typical example, MicroRNA‐34a (miR34a) serves as a tumor suppressor in glioma, which is associated with tumor prognosis.^19^ Also, the level of sex‐determining region Y‐box 2 (SOX2), CD44, Notch, which is strongly related to the self‐renew and chemoresistance of GICs, was modulated by miR34a expression.^20^ Therefore, miR34a might serve as a powerful weapon against GICs, which would conquer the multiple signaling pathways associated with GICs stemness and drug resistance. Accordingly, miR34a was here applied as the model RNAi drug.

**Scheme 1:**
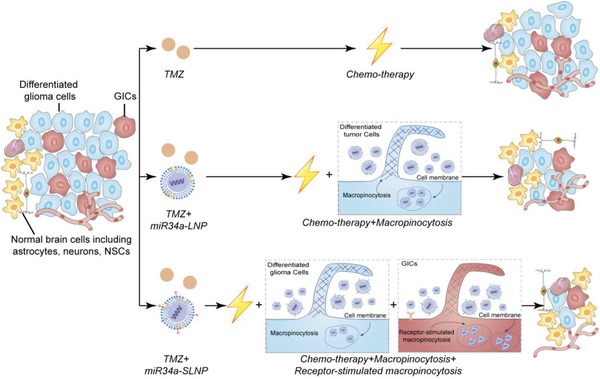
SLNPs enable efficient targeting miRNA delivery to GICs via receptor‐stimulated micropinocytosis to efficiently inhibit self‐renewal and chemoresistance.

Delivering microRNA into the GICs faces the challenge. To realize efficient microRNAs delivery to GICs through the CXCR4‐stimulated macropinocytosis pathway, a drug‐delivery system for efficient microRNAs loading and release is essential. Lipoproteins, natural nanostructures possessing favorable surface properties with an extended period in the circulation, are suitable for delivering therapeutic agents. It could protect the microRNAs in the circulation and deliver microRNAs to the recipient cells.^21^ Here, to achieve high microRNA loading and to realize cytoplasm drug release, calcium phosphate (CaP) was incorporated into the core of the lipoprotein nanoparticles as described previously.^22^ An α‐helix sequence (Ac‐FAEKFKEAVKDYFAKFWD), which mimics the binding effect of apolipoprotein and exhibits strong lipid‐association properties, was employed to assemble the SDF1 mimic peptide into the lipoprotein nanoparticle.^23^ The synthesized tailored nanoparticle was named as stimulated lipoprotein‐like nanoparticles (SLNPs). As expected, the tailored lipoprotein‐like nanostructure achieved highly efficient GICs‐targeting accumulation via CXCR4‐stimulated macropinocytosis. With miR34a loading, the nanoformulation efficiently inhibited the self‐renewal and chemoresistant ability in GICs and sharply prolonged the animal survival in GICs derived orthotopic mice models safely, especially when combined with chemotherapeutics (**Scheme** **1**).

## 2 Results and Discussion

### 2.1 Preparation and Characterizations of MiRNA‐Loaded SLNPs

**Figure 1:**
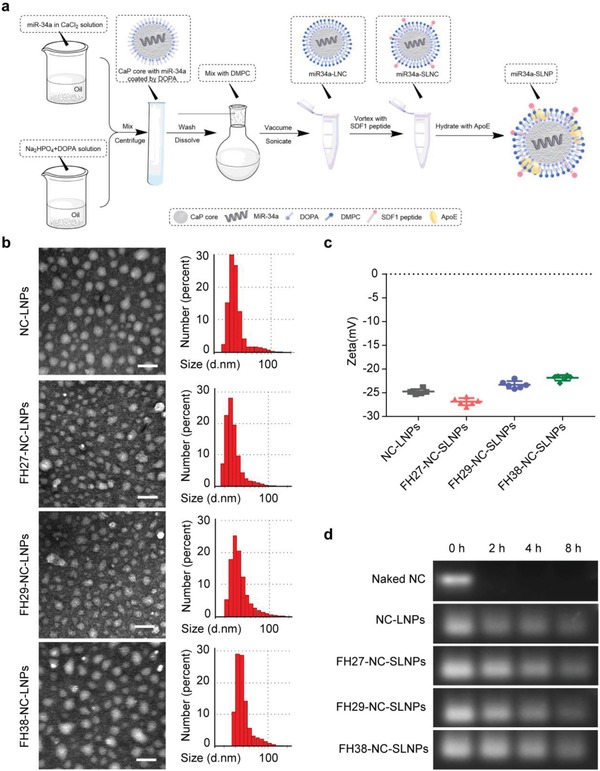
Preparation and characterization of NC‐SLNPs. a) The outline for the preparation of SLNPs nanoparticles loaded with microRNA. b) Morphology and particle size distribution of NC‐SLNPs (FH27/FH29/FH38) under a transmission microscope and dynamic light scattering. Scale bar, 50 nm. c) Particle zeta potential of NC‐SLNPs (FH27/FH29/FH38). d) Serum stability of miRNA loaded by LNPs and SLNPs at different incubation times.

The SLNPs loading miRNA were developed through the approach as shown in **Figure** **1**a, miRNA‐loaded CaP core was prepared through the water‐in‐oil microemulsion method.[qv: 22b] Dioleoylphosphatydic acid (DOPA) was then applied to coat on the surface of CaP core. Subsequently, 1,2‐dimyristoyl‐sn‐glycerol‐3‐phosphocholine (DMPC) was employed to encapsulate the CaP core that loaded the negative control (NC) miRNA and miR34a. The resulting nanoparticles were named as NC miRNA‐loaded lipid nanocarrier (NC‐LNC) and miR34a‐loaded lipid nanocarrier (miR34a‐LNC), respectively. Hybrid peptides containing an α‐helix (Ac‐FAEKFKEAVKDYFAKFWD) sequence, a glycine‐serine‐glycine peptide linker, and synthetic SDF1 mimic peptides were synthesized to assemble the SDF1‐mimic peptides to the surface of SLNPs. The hybrid peptides enclosing the binding motif of SDF1 (RFFESH), the activation motif of SDF1 (KPVSLSYR), and the binding and activation motif of SDF1 (KPVSLSYR‐APA‐RFFESH) was named as FH27, FH29, and FH38, respectively. Specifically, in the sequence of FH38 peptide, the native SDF1 cysteine‐proline‐cysteine linker was changed to alanine‐proline‐alanine (APA) to reduce its chemotactic activity and avoid the induction of cancer cells migration.[qv: 16b] MiR34a‐SLNPs were then assembled via a two‐step incubation procedure: miR34a‐LNC was incubated with the FH27, FH29, and FH38 peptides at the ratio of 1:100 (peptides: DMPC, molar ratio) for 24 h to form the SDF1‐mimic peptide‐incorporated LNC, named as stimulated LNC (SLNC) (FH27‐miR34a‐SLNC, FH29‐miR34a‐SLNC, and FH38‐miR34a‐SLNC). After that, the nanocomplex was then incubated with ApoE3 at the ratio of 1:8 (ApoE3: DMPC, w/w) for another 24 h to obtain FH27‐miR34a‐SLNPs, FH29‐miR34a‐SLNPs, and FH38‐miR34a‐SLNPs, respectively. The nanocomplex loaded with negative control miRNA and miR34a, with ApoE3 but without SDF1‐mimic peptide, was named as NC‐LNP and miR34a‐LNP, respectively. 1,1′‐Dioctadecyl‐3,3,3′,3′‐tetramethyl indocarbocyanine perchlorate (DiI), a fluorescent probe widely used for liposome labeling, was incorporated into the nanoparticles for fluorescent imaging.

Dynamic light scattering (DLS) and transmission electronic microscope (TEM) were used to characterize the nanoparticles. The sizes of the miRNA‐loaded nanocomplex were in the range of 20–45 nm. Under TEM, NC‐LNPs exhibited compact and spherical morphology (25–30 nm). FH27‐NC‐SLNPs and FH29‐NC‐SLNPs exhibited more rough surface (20–35 nm) and FH38‐NC‐SLNPs showed a slightly bigger size compared with NC‐LNPs (35–45 nm) (Figure 1b). The polydispersity index of the nanocomplex was between 0.2 and 0.4, demonstrating a low‐to‐moderate distribution of size. The zeta potentials of miRNA‐SLNPs were all negative, but slightly different due to the surface modification: FH27‐miR34a‐SLNPs were shown more negative (−26.8 ± 1.2 mV), and FH29‐miR34a‐SLNPs (−23.3 ± 0.9 mV) and FH38‐miR34a‐SLNPs (−21.8 ± 1.1 mV) were slightly more positive than NC‐LNPs (−24.7 ± 0.6 mV) (Figure 1c). The encapsulation efficiency of miRNA in SLNPs was 45.5% ± 3.5%. The loading capacity of miRNA in the SLNPs was 1.21 ± 0.09% w/w. To evaluate the capacity of SLNPs as carriers for intracellular microRNA delivery, 50% serum was here applied to provide a harsh condition to evaluate the stability of miRNA. Naked miRNA was found totally degraded after only 2 h incubation in 50% serum, while 30–35% miRNA in SLNPs was degraded after 4 h incubation and 45–50% after 8 h incubation (Figure 1d), suggesting that the encapsulation of miRNA in SLNPs can efficiently protect miRNA from degradation.

### 2.2 Patient‐Derived GICs Efficiently Captured SLNPs via CXCR4‐Stimulated Macropinocytosis

**Figure 2:**
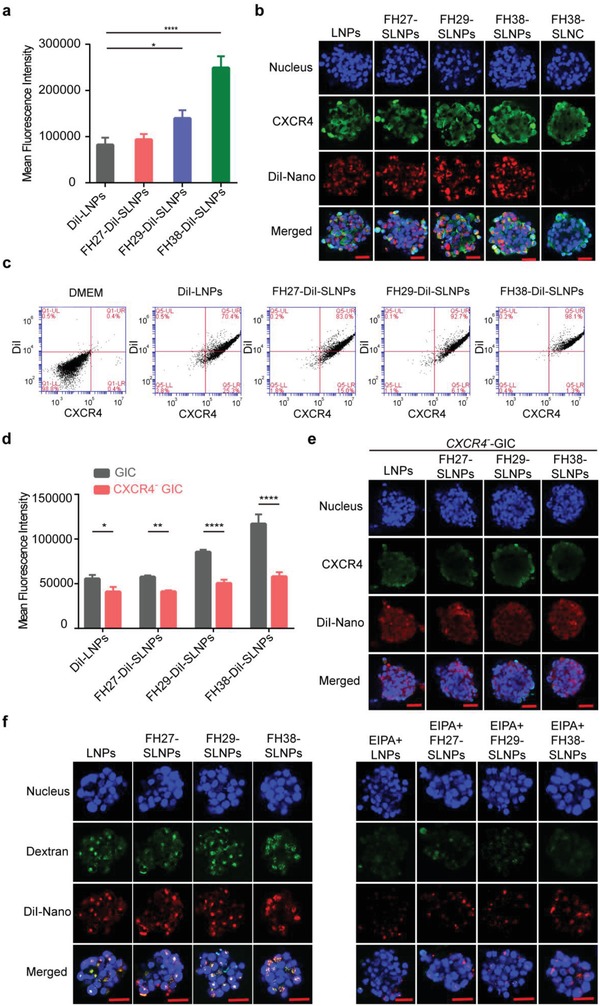
Patient‐derived GICs efficiently captured SLNPs via CXCR4‐stimulated macropinocytosis. a) SDF1 mimic peptides (FH27/FH29/FH38) modification enhanced the cellular uptake of DiI‐labeled SLNPs. SLNPs were prepared at the peptides: DMPC, molar ratio 1:100, and incubated with GICs at 37 °C for 3.5 h at the DMPC concentration of 20 µg mL^−1^ (*n* = 3). The significance of the differences was evaluated by one‐way ANOVA followed by Bonferroni test (*p < 0.05, ****p < 0.0001). b) Cellular uptake of DiI‐SLNPs and colocalization of SLNPs to CXCR4. Scale bar, 100 µm. c) The FH38‐DiI‐SLNPs showed the highest percentage of colocalization with CXCR4. d) Knockdown of CXCR4 led to a reduction in the cellular uptake of DiI‐LNPs and DiI‐SLNPs (*n* = 3). The significance of the differences between two groups (**p < 0.01, ****p < 0.0001) was evaluated by two‐tailed Student's *t*‐test. e) Qualitative analysis of the GICs uptake of LNPs and SLNPs after knocking down CXCR4. Scale bar, 100 µm. f) Colocalization of DiI‐LNPs and DiI‐SLNPs to macropinocytosis marker FITC‐70 kDa dextran in GICs in the absence/presence of EIPA (150 × 10^−6^ m). Scale bar, 100 µm.

GICs are extremely hard to control. Extensive endeavor including targeting specific biomarkers and signaling, regulating microenvironment, cell membrane based targeting therapeutics, targeting immunotherapies has been made for the management of TICs.^24^ As GICs, differentiated cancer cells and normal counterparts exhibit different macropinocytosis dependencies, here the upregulated internalization efficiency of developed SLNPs by self‐amplified macropinocytosis manner was observed. To evaluate the GICs targeting effect, patient‐derived GICs were applied as one of the tumor models. The self‐renewal features of GICs in vitro had been characterized as previously reported.[qv: 22b] To further characterize the phenotype of the patient‐derived GICs in vivo, here we further stained the patient tumor sample, and the GICs‐derived xenograft sample with Hematoxylin and eosin, anti‐CD44, anti‐SOX2, and anti‐PTEN (gene of phosphate and tension homology deleted on chromsome ten) antibodies. It was found that the GICs‐derived xenograft samples showed similar phenotype with patient tumor sample, demonstrating the similar strongly positive expression of CD44 and SOX2 and negative expression of PTEN (Figure S1, Supporting Information). In this GICs model, more highly efficient and time‐dependent accumulation of FH38‐DiI‐SLNPs compared with DiI‐LNPs, was monitored by a live‐cell imaging system (Movie S1, Supporting Information). Quantitative analysis showed that the GICs absorption of FH38‐DiI‐SLNPs was much higher (threefold) than that of DiI‐LNPs, while the uptake of FH27‐DiI‐SLNPs (1.1‐fold) was comparable with DiI‐LNPs, and that of FH29‐DiI‐SLNPs was slightly higher (1.7‐fold) (**Figure** **2**a).

To determine whether the enhanced internalization of SLNPs was CXCR4 associated, we first analyzed the colocalization between SLNPs and CXCR4. Laser confocal imaging showed that FH38‐DiI‐SLNPs were internalized into the GICs and colocalized with the CXCR4 receptor (Figure 2b). The Pearson's correlation coefficient values indicated that FH38‐DiI‐SLNPs (0.61) displayed higher colocalization with CXCR4 than FH27‐DiI‐SLNPs (0.48), FH29‐DiI‐SLNPs (0.43), and DiI‐LNPs (0.41) (Figure S2a, Supporting Information). Flow cytometry analysis confirmed that 95–98% of GICs were FH38‐DiI‐SLNPs and CXCR4 double‐positive, while 85–92% of GICs were FH29‐DiI‐SLNPs and CXCR4 double‐positive, 83–93% of GICs were FH27‐DiI‐SLNPs and CXCR4 double‐positive, and 70–78% of GICs were DiI‐LNPs and CXCR4 double‐positive (Figure 2c and Figure S2b, Supporting Information). This could be resulted from the different sequence and function of the mimic peptides, in which FH27 and FH29 only exhibit partial functions of SDF1, while FH38 peptide contains the SDF1 sequences responsible for both the binding and the activation activity toward CXCR4. It was also found that the incorporation of ApoE played a crucial role in facilitating cellular uptake of the nanoparticles. The incubation of LNCs with ApoE3 reduced both the size and zeta potential of LNC, which could be due to the interaction between lipid and the negative charged ApoE3. More importantly, the incorporation of ApoE3 largely enhanced the cellular internalization of the nanoparticles in GICs (Figure S3, Supporting Information). It is because that ApoE3 could play as the protein nutrient for Ras‐activated tumor cells to “eat.”[qv: 22b] It was also observed that higher loading ratio of FH38 peptide led to the more efficient internalization of FH38‐SLNPs (Figure S4, Supporting Information), confirming that the SDF1‐mimic peptides are critical to the enhanced cellular uptake of SLNPs in GICs. These evidence collectively indicated the involvement of CXCR4 in the enhanced cellular uptake of SNLPs in GICs.

To determine whether SLNPs were internalized in GICs through the CXCR4‐enhanced macropinocytosis as designed, we further evaluated the uptake of SLNPs in GICs when CXCR4 expression was knocked down by lentivirus‐mediated anti‐CXCR4 short hairpin RNA (shRNA) and macropinocytosis was inhibited by specific inhibitor ethylisopropylamiloride (EIPA). CXCR4 knockdown significantly reduced the cellular association of all the three SLNPs (Figure 2d,e and Figure S5, Supporting Information). CXCR4 knockdown also slightly reduced the cellular uptake of LNPs, which could be because that CXCR4 is partially involved in the macropinocytosis pathway.[qv: 12b] Nevertheless, knockdown of CXCR4 induced much higher extent of reduced cellular association of SLNPs, suggesting that CXCR4‐stimulated macropinocytosis plays an important role in the cellular uptake of SLNPs. In addition, confocal microscopy found that macropinocytosis marker fluorescein isothiocyanate (FITC)‐70 kDa dextran (green) was highly colocalized (yellow) with DiI‐SLNPs (red). The 70 kDa dextran is the specific probe that could be internalized into macropinosomes generated by the ruffling of macropinocytosis progress.[qv: 5a,25] The Pearson's correlation coefficient values of SLNPs and dextran were all above 0.8 in GICs model (Figure S6, Supporting Information). Moreover, macropinocytosis inhibitor EIPA significantly decreased the internalization of all the three SLNPs in GICs (more than 65%) (Figure 2f). These evidence collectively indicated that SLNPs achieved specific and enhanced accumulation in GICs through the CXCR4‐stimulated macropinocytosis.

As CXCR4 is also highly expressed in normal neural stem cells (NSCs), it needs to be considered whether SLNPs could also be captured by NSCs. It was found that the NSCs derived from the subventricular zone of mice brain could capture all the nanoparticles (DiI‐LNPs, FH27‐SLNPs, FH29‐SLNPs, and FH38‐SLNPs), but without any difference and at much lower levels than that in GICs (Figure S7, Supporting Information). A FH38‐SLNP did not significantly arise receptor‐mediated macropinocytosis in NSCs as it did in GICs. The underlying mechanisms of specific CXCR4‐stimulated macropinocytosis in GICs could be attributed to the fact that macropinocytosis provides not only a survival mechanism under nutrient‐deficient conditions but also the potential for unrestricted tumor growth in an adverse tumor microenvironment.^26^ Actually, macropinocytosis is found to support tumor cell growth under nutrient‐limiting conditions through Ras activation.^5^ In the case of neural stem cells and other normal cells in the brain, where the nutrient is sufficient,^27^ the macropinocytosis pathway might not be as important as that in the GICs. Therefore, the CXCR4‐stimulated macropinocytosis can be utilized as a specific mechanism to target GICs.

### 2.3 FH38‐SLNPs Efficiently Targeted GICs In Vivo in a CXCR4‐Dependent Manner

**Figure 3:**
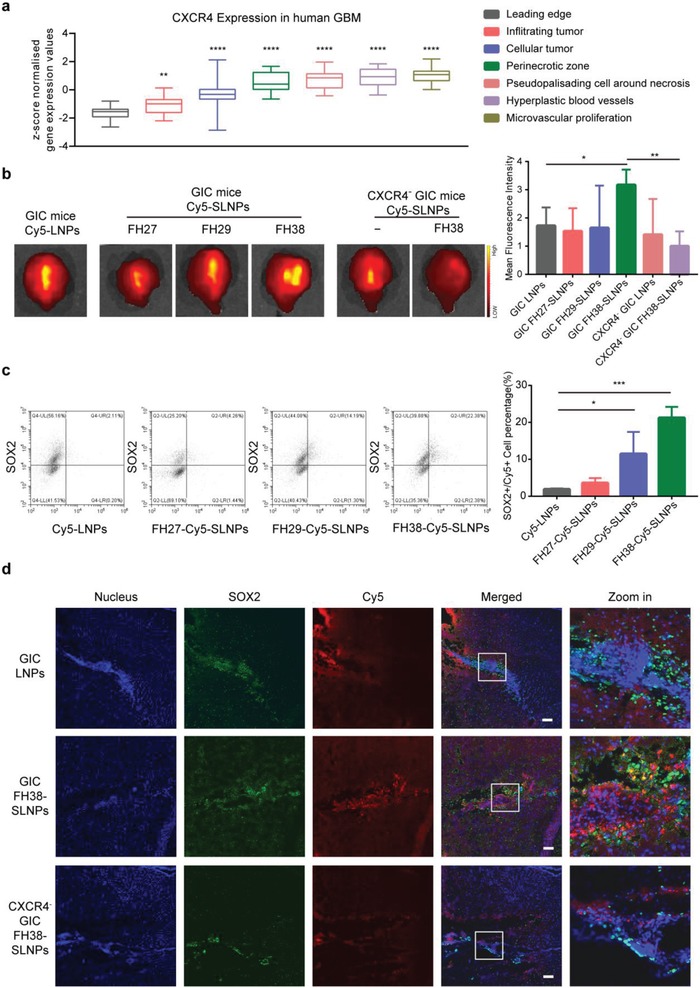
Enhanced GICs‐targeting efficiency of SLNPs in vivo in a CXCR4‐dependent manner. a) CXCR4 expression was evaluated in the different histological locations of human GBM samples. b) Qualitative analysis and semi‐qualitative analysis of tumor accumulation of Cy5‐LNPs and Cy5‐SLNPs at 4 h postinjection in the mice bearing GICs‐derived glioma and CXCR4 knocked down GICs‐derived glioma (*n* = 3). c) Quantitative analysis of SOX2 positive GICs colocation of Cy5‐LNPs and Cy5‐SLNPs at 4 h postinjection in the mice bearing GICs‐derived tumor. d) Brain distribution of Cy5‐LNPs and FH38‐Cy5‐SLNPs at 4 h postinjection in the mice bearing GICs‐derived glioma and CXCR4 knocked down GICs‐derived glioma (*n* = 3). Scale bar, 100 µm. For (b) and (c), the significance of the differences was evaluated by one‐way ANOVA followed by Bonferroni test (*p < 0.05, **p < 0.01, ***p < 0.001).

To confirm the association between CXCR4 and GICs, we analyzed the expression of CXCR4 in human glioblastoma multiforme (GBM) samples through the RNA‐sequencing data (*n* = 19–111) obtained from the Ivy Glioma Atlas Project (**Figure** **3**a). CXCR4 expression in the different histological locations including the leading edge (LE), infiltrating tumor, cellular tumor (CT), perinecrotic zone (PZ), cellular tumor‐pseudopalisading cells around necrosis (CT‐PAN), cellular tumor‐hyperplastic blood vessels (CT‐HBV) and cellular tumor‐microvascular proliferation (CT‐MVP) of GBM was evaluated. It has been demonstrated that GICs were found in the vascular regions and hypoxic regions. Interestingly, the expression profile of CXCR4 was correlated to the distribution of GICs. Higher CXCR4 expression was found in those regions associated with angiogenesis such as CT‐HBV, CT‐MVP, or related to pseudopalisading necrosis such as PZ, CT‐PAN, compared with those regions with only one to three tumor cells such as LE. Moreover, CXCR4 signaling pathway would result in downstream macropinocytosis stimulation. Therefore, CXCR4 was confirmed as an ideal target for GICs‐targeted drug delivery.

To evaluate the GICs‐targeting capability of SLNPs in vivo, we used Cy5‐labeled negative control miRNA as the cargo and indicator. Thirty days after intracranial implantation with GICs and CXCR4‐knockdown GICs in nonobese diabetic/severe combined immunodeficient (NOD/SCID) mice, FH38‐Cy5‐SLNPs achieved the highest accumulation at the tumor site at 4 h postinjection among the three types of SLNPs. In concert with in vitro observation, CXCR4 knockdown significantly reduced accumulation of FH38‐Cy5‐SLNPs at the tumor sites (Figure 3b). Flow cytometry measurement was used to quantify the colocation of SOX2 positive GICs and Cy5‐LNPs/Cy5‐SLNPs, finding that FH38‐Cy5‐SLNPs group achieved the highest percentage of Cy5 and SOX2 double‐positive cell population (20%), while FH27‐Cy5‐SLNPs group achieved 4%, FH29‐Cy5‐SLNPs group achieved 14%, and LNPs group showed the lowest 2% (Figure 3c). For all the SOX2‐positive cells, the FH38‐Cy5‐SLNPs captured 30–36% of the cells, while Cy5‐LNPs captured only 3–4%, FH27‐Cy5‐SLNPs captured 7–15%, and FH29‐Cy5‐SLNPs captured 7–19%. Moreover, confocal imaging of the brain frozen sections showed that compared with Cy5‐ LNPs, FH38‐Cy5‐SLNPs more effectively accumulated at the SOX2‐positive GICs sites. But when CXCR4 was knocked down, FH38‐Cy5‐SLNPs no longer showed specific accumulation at the SOX2‐positive GICs sites (Figure 3d). Altogether, these results suggested that FH38‐SLNPs could cross the blood–brain barrier, specifically accumulate into the SOX2‐positive GICs in vivo in a CXCR4‐dependent manner, and serve as a powerful nanoplatform for GICs‐targeting drug delivery.

### 2.4 MiR34a‐SLNPs Inhibited the Self‐Renewal and Chemoresistant Capacity of GICs

Given that SLNPs can efficiently target GICs through enhanced macropinocytosis, we then used it for delivering microRNAs to GICs to inhibit multiple signaling pathways that associate with self‐renewal and chemoresistant ability. For efficient miRNA delivery, the intracellular release of miRNA from the nanoparticles and pinocytotic vesicles is crucial. To clearly determine the intracellular fate of miRNA in GICs, the spheres were dissociated into single cells suspension. As shown in Figure S8 in the Supporting Information, with the increase of the incubation time, increasing fluorescence of carboxyfluorescein (FAM)‐NC was found to escape from the lysosome and release into the cytoplasm of GIC cells. The intracellular dissociation of miRNA from the carrier was also found to be a time‐dependent process. Especially, FH27‐SLNPs and FH38‐SLNPs showed higher levels of intracellular miRNA release compare with FH29‐SLNPs, which could be induced by the different functions of the peptides.

**Figure 4:**
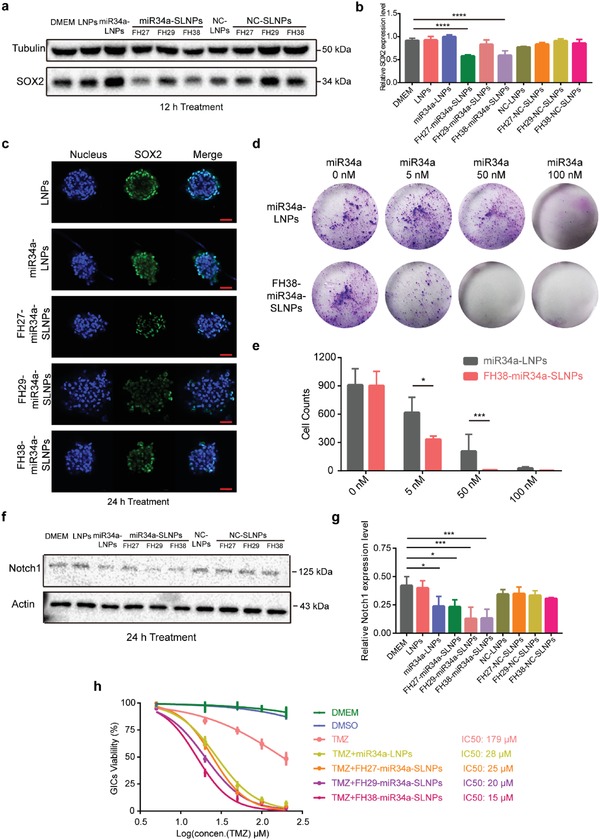
miR34a‐SLNPs inhibited the self‐renewal and reversed temozolomide resistance of GICs. a,b) The protein level of SOX2 in GICs after treatment with NC‐LNPs, NC‐SLNPs, miR34a‐LNPs, and miR34a‐SLNPs at the miRNA concentration 100 × 10^−9^ m for 12 h. c) Laser confocal imaging of the SOX2 protein expression in GICs after treatment with miR34a‐LNPs and miR34a‐SLNPs at the miRNA concentration 100 × 10^−9^ m for 24 h. Scale bar, 50 µm. d) Representative images and e) quantitative analysis of in vitro colony formation assay of GICs treated with miR34a‐LNPs and FH38‐miR34a‐SLNPs for 48 h (*n* = 3). The significance of the differences between two groups (*p < 0.05, ***p < 0.001) was evaluated by two‐tailed Student's *t*‐test. f,g) The protein level of Notch1 in GICs after treatment with NC‐LNPs, NC‐SLNPs, miR34a‐LNPs, and miR34a‐SLNPs at the miRNA concentration 100 × 10^−9^ m for 24 h. h) Cell viability of GICs after treatment with NC‐LNPs, miR34a‐LNPs, and miR34a‐SLNPs at the concentration of 50 × 10^−9^ m miR34a combination with the different concentration of TMZ (5 × 10^−6^, 20 × 10^−6^, 50 × 10^−6^, 100 × 10^−6^, and 200 × 10^−6^ m) for 24 h. For (b) and (g), data represent mean ± s.d. (*n* = 3). The significance of the differences was evaluated by one‐way ANOVA followed by Bonferroni test (*p < 0.05, ***p < 0.001, ****p < 0.0001).

After that, we continued to evaluate the effect of MiR34a‐SLNPs on GICs. SOX2 exerts a crucial role in the maintenance of an undifferentiated state of GICs, serving as an essential anti‐GICs target.^28^ It was found that following the treatment with miR34a‐LNPs for 12 h at 100 × 10^−9^ m miR34a, the expression level of SOX2 in GICs was similar with that in those GICs treated with dulbecco's modified Eagle media (DMEM). But following the treatment with FH27‐miR34a‐SLNPs, FH29‐miR34a‐SLNPs, and FH38‐miR34a‐SLNPs at the same miR34a concentration, the expression level of SOX2 was reduced by 34%, 10%, and 35% (**Figure** **4**a,b). The relatively higher efficiency of FH27‐miR34a‐SLNPs than FH29‐miR34a‐SLNPs for SOX2 knockdown could be caused by the higher level of intracellular miRNA release of FH27‐miR34a‐SLNPs (Figure S8, Supporting Information). Such finding was confirmed by confocal imaging analysis for a 24 h treatment (Figure 4c). Clone counting was then used to further verify the inhibition effect of miR34a‐SLNPs on GICs self‐renewal ability. Compared with the miR34a‐LNPs‐treated GICs group, the FH38‐miR34a‐SLNPs‐treated group formed significantly fewer and smaller colonies in the miR34a concentration range of 5 × 10^−9^–50 × 10^−9^ m (Figure 4d,e). This tailored nanoparticle inhibited GICs more efficiently, compared with other gene delivery systems in which more than 100 × 10^−9^ m miR34a was needed to control GICs.^29^

Besides stemness, the resistance of GICs to chemotherapy plays a critical role in cancer propagation. Notch signaling pathway is one of the most important route contributing to chemoresistance in GICs.^30^ MiR34a can inhibit multiple pathways including the chemoresistant‐related Notch1 signaling. In clinic, temozolomide (TMZ) resistance is common in GBM therapy, in which Notch1 signaling is also involved.^31^ Here we found that all the miR34a‐SLNPs efficiently silenced Notch1 after 24 h treatment. Especially, FH38‐miR34a‐SLNPs achieved the highest silencing efficiency and reduced Notch1 expression by 62% (Figure 4f,g). It was demonstrated that the IC50 values of TMZ‐sensitive glioma stem cells may range from 40 × 10^−6^ to 100 × 10^−6^ m, while the IC50 of relative TMZ‐resistant glioma cells range from 120 × 10^−6^ to 200 × 10^−6^ m.^32^ All the miR34a formulations significantly reduced the IC50 of TMZ, among which FH38‐miR34a‐SLNPs were the most efficient, reducing the IC50 of TMZ from 179 × 10^−6^ to 15 × 10^−6^ m (Figure 4h). The combination of TMZ and miR34a‐SLNPs also powerfully damaged the GICs spheres (Figure S9, Supporting Information). Collectively, the above results clearly demonstrated the potent therapeutic effect of miR34a‐SLNPs on the inhibition of self‐renewal and chemoresistance of GICs.

### 2.5 MiR34a‐SLNPs Efficiently Suppressed Tumor Growth and Prolonged Animal Survival in GICs‐Derived Orthotopic Mice Models

**Figure 5:**
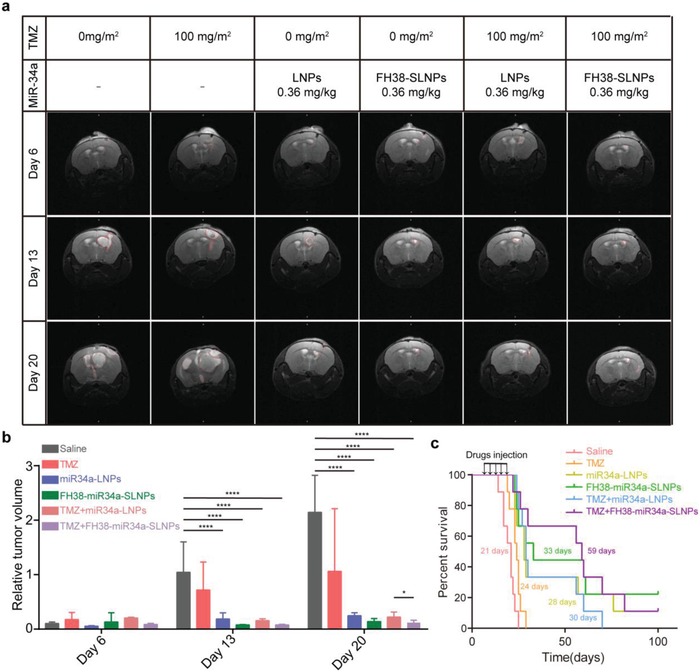
miR34a‐SLNPs efficiently suppressed tumor growth and prolonged animal survival in GICs‐derived orthotopic mice models. a) MR imaging of intracranial GICs derived tumors. The images were collected before the initiation of treatment (Day 6) and during the procedure (Day 13 and Day 20). The red lines indicate the probable outline of the brain tumors. b) The relative tumor volume was quantified from the MR images. The significance of the differences was evaluated by one‐way ANOVA followed by Bonferroni test (*p < 0.05, ****p < 0.0001). c) Kaplane Meier survival curve of mice bearing patient‐derived GICs glioma treated with saline, TMZ, miR34a‐LNPs, FH38‐miR34a‐SLNPs, TMZ+miR34a‐LNPs, or TMZ+ FH38‐miR34a‐SLNPs every 3 d for five times at microRNA dose of 0.36 mg kg^−1^ (*n* = 9).

Finally, we assessed the potential of miR34a‐SLNPs for the therapy against glioma cancer in vivo. Since FH38‐SLNPs showed the best targeting efficiency among the three types of SLNPs, here we evaluated the therapeutic effect of FH38‐miR34a‐SLNPs. In mice model raised from patient‐derived GICs, miR34a‐LNPs and FH38‐miR34a‐SLNPs were given intravenously at the miR34a dosage of 0.36 mg kg^−1^ on Day 7, 10, 13, 16, and 19 after GICs inoculation. At the same time, TMZ was given via oral gavage at the dose of 100 mg m^−2^. Magnetic resonance imaging (MRI) analysis showed that compared with those animals treated with saline or TMZ alone, remarkable blocking of tumor growth was observed in the miR34a‐LNPs, FH38‐miR34a‐SLNPs, TMZ+miR34a‐LNPs, and TMZ+FH38‐miR34a‐SLNPs treatment groups. Especially on Day 20, the TMZ+FH38‐miR34a‐SLNPs treatment significantly suppressed the tumor volume compared with TMZ+miR34a‐SLNPs treatment, indicating the better therapeutic effect of FH38‐miR34a‐SLNPs than miR34a‐SLNPs (**Figure** **5**a,b). Consistent with the MRI results, the mean survival of mice bearing GICs glioma given with saline, TMZ, miR34a‐LNPs, FH38‐miR34a‐SLNPs, TMZ+miR34a‐LNPs, and TMZ+FH38‐miR34a‐SLNPs were 21, 24, 28, 33, 30, and 59 d, respectively (Figure 5c). Noticeably, 22% of mice receiving FH38‐miR34a‐SLNPs and 11% of mice receiving TMZ+FH38‐miR34a‐SLNPs survived more than 100 d. FH38‐miR34a‐SLNPs/TMZ+FH38‐miR34a‐SLNPs  showed significantly better therapeutic effect than miR34a‐LNPs/TMZ+miR34a‐LNPs, which indicated that the higher suppression efficiency of GICs could promote the better anticancer efficacy. In addition, one important point in the patient‐derived GICs tumor models is that miR34a‐LNPs+TMZ treated mice showed similar survival with that of the miR34a‐LNPs treated animals, while FH38‐miR34a‐SLNPs+TMZ treatment significantly improved survival compared with simple FH38‐miR34a‐SLNPs treatment. These data suggested that in the presence of chemotherapy, FH38‐miR34a‐SLNPs captured GICs more efficiently, which could overcome chemoresistance more powerfully. In contrast, MiR34a‐LNPs, which exhibited relative lower GICs‐targeting efficiency cannot inhibit the drug‐resistant GICs as efficiently as FH38‐miR34a‐SLNPs. So that there was no difference in the antitumor efficacy between miR34a‐LNPs and TMZ+miR34a‐LNPs. Compared with our previous work in which ATF5‐CaP‐rHDL prolonged the median survival of mice bearing GICs for 40 d, FH38‐miR34a‐SLNPs combined TMZ prolonged the median survival for 59 d in the same mice model.[qv: 22b] Collectively, these data provided direct evidence to support our hypothesis that FH38‐miR34a‐SLNPs could much more powerfully combat malignancies through the more efficient control of GICs.

**Figure 6:**
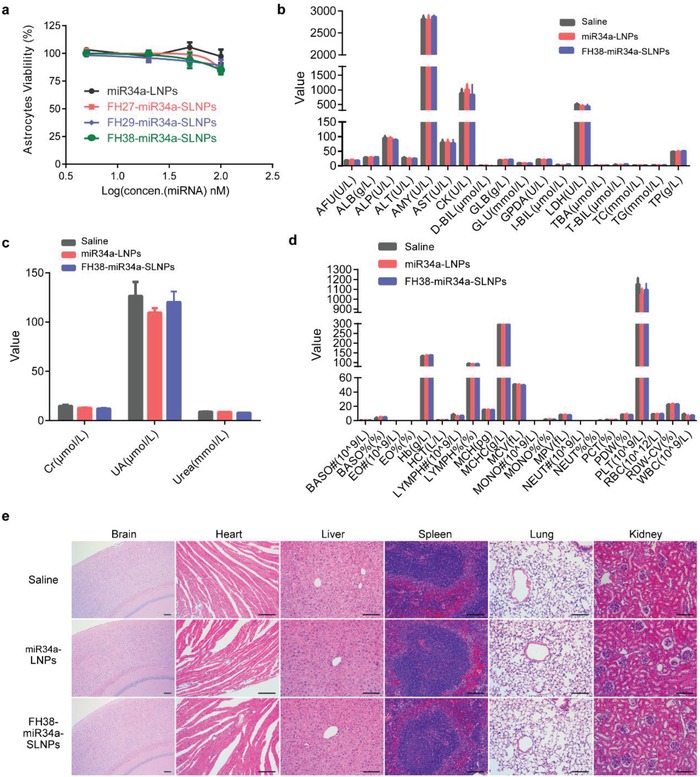
Safety of miR34a‐LNPs and FH38‐MiR34a‐SLNPs on healthy ICR mice. a) Cell viability of the primary astrocytes after the treatment with miR34a‐LNPs, FH27‐miR34a‐SLNPs, FH29‐miR34a‐SLNPs, and FH38‐miR34a‐SLNPs. b–d) Healthy ICR mice were intravenously treated with saline miR34a‐LNPs or FH38‐miR34a‐SLNPs at the siRNA dose of 0.36 mg kg^−1^ every 3 d for totally five doses (*n* = 4). Blood biochemistry and hematology tests were performed at 1 d after the last injection. AFU: alpha‐L‐fucosidase, ALB: albumin, ALP: alkaline phosphatase, ALT: alanine aminotransferase, AMY: amylase, AST: aspartate transaminase, CK: creatine kinase, DBIL: direct bilirubin, GLB: globulin, GLU: glucose, GPDA: glycyl proline dipeptidyI aminopeptidase, IBIL: indirect bilirubin, LDH: lactate dehydrogenase, TBA: thiobarbituric acid, TBIL: total bilirubin, TC: total cholesterol, TG: triglyceride, TP: total protein, CR: creatinine, UREA: urease, UA: uric acid, BASO: basophils, EO: eosinophils, HB: hemoglobin, HCT: hematocrit, LYMPH: lymphocytes, MCH: mean corpuscular hemoglobin, MCHC: mean corpuscular hemoglobin concentration, MCV: erythrocyte mean corpuscular volume, MONO: monocytes, MPV: mean platelet volume, NEUT: neutrophils, PCT: procalcitonin, PDW: platelet distribution width, PLT: platelet, RBC: red blood cell, RDW‐CV: red cell distribution width (coefficient of variation), WBC: white blood cell. Data represent mean ± s.d. Unpaired student's *t*‐test (two tailed) was used for comparison between two groups and *p* < 0.05 were considered significant. e) Hematoxylin and eosin staining of the major organs. Scale bar, 50 µm.

Besides efficacy, safety is another important concern. The potential toxicity of miR34a‐LNPs and FH38‐miR34a‐SLNPs was additionally analyzed in normal mice following the same administration procedure. After the treatment, blood chemistry and morphological observation were carried out and no notable changes were observed among the mice treated with saline, miR34a‐LNPs, and FH38‐miR34a‐SLNPs (**Figure** **6**). These results collaboratively demonstrated the safety of miR34a‐LNPs and FH38‐miR34a‐SLNPs.

## 3 Conclusion

In summary, we developed a CXCR4 receptor‐stimulated lipoprotein‐like nanoparticle to amplify their internalization into GICs through receptor‐stimulated macropinocytosis. We analyzed the CXCR4 stimulated effect of three peptides, which mimic the functions of SDF1. Among the three SDF1 mimic peptides for specific stimulating macropinocytosis in GICs, we found that FH38 was the most efficient for the accumulation effect in GICs both in vitro and in vivo. Moreover, the miR34a drugs loaded by FH38‐modified nanoparticles efficiently suppressed the stemness and chemoresistance through blocking SOX2 and Notch1 expression at relatively low dosage. It thus promoted the therapeutic effect of chemodrugs and prolonged the survival of GICs‐bearing mice model with negligible toxicity. Therefore, these data provide proof‐of‐concept evidence for the design of highly efficient nanoplatform for GICs‐targeting therapy through stimulated macropinocytosis, which also highlight the potential of precision therapeutics for combating tumor malignancies.

## 4 Experimental Section

### Materials

1,2‐Dioleoyl‐*sn*‐glycero‐3‐phosphate (sodium salt) (DOPA) and 1,2‐dimyristoyl‐*sn*‐glycero‐3‐phosphocholine (DMPC) were obtained from Avanti Polar Lipids (Alabaster, AL, USA). Cyclohexane and IGEPAL CO‐520 were provided by Sigma‐Aldrich (St Louis, MO, USA). Recombinant Human ApoE3 was synthesized by PEPROTECH (Rocky Hill, NJ, USA). All microRNAs used both in vitro and in vivo were synthesized from Ribobio (Guangzhou, China). The miR34a consisted of the antisense strand 5′‐ACAACCAGCUAAGACACUGCCA‐3′ and sense strand 5′‐UGGCAGUGUCUUAGCUGGUUGU‐3′. NC‐miRNAs have an antisense strand 5′‐CAGUACUUUUGUGUAGUACAAA‐3′ and sense strand 5′‐UUUGUACUACACAAAAGUACUG‐3′. The miRNAs labeled with fluorescent dye such as Cy5 on the antisense strand 5′ helped for imaging and tracking. SOX2 rabbit polyclonal antibody (ab97959), CXCR4 rabbit monoclonal antibody (ab181020) and Notch1 rabbit monoclonal antibody (ab52627) were purchased from Abcam (Cambridge, UK). DiI was obtained from Invitrogen (Carlsbad, CA, USA). Cell counting kit‐8 (CCK8) was provided by Dojindo Laboratories (Kumamoto, Japan). Other reagents were obtained from Sigma‐Aldrich if not specifically mentioned.

### Cells

Glioma specimens were obtained from the surgery of a 4‐year‐old patient at the Department of Neurosurgery in Shanghai Renji Hospital (Shanghai, China) with appropriate consent from guardians and in accordance with the ethics committee approved protocol. GICs were derived from the tumor samples as described previously.[qv: 22b] Briefly, samples were minced and disaggregated by compound‐enzyme with 0.05% Trypsin‐Ethylene diamine tetraacetic acid (EDTA) and Type 4 Collagenase. Isolated cells were cultured at a relatively low density (1≈3 × 10^5^ cells mL^−1^) in serum‐free medium supplementing growth factors (20 ng mL^−1^ bFGF and 20 ng mL^−1^ EGF). The neural stem cells were obtained from the subventricular zone of the 8‐week‐old mice in accordance with the guidelines approved by the Animal Experimentation Ethics Committee of Shanghai Jiao Tong University School of Medicine. Briefly, the tissue of subventricular zone was dissociated and cultured in Neural basal medium supplementing growth factors (20 ng mL^−1^ bFGF and 10 ng mL^−1^ EGF).^33^

### Establishment of GICs‐Derived Orthotopic Mice Models

The animal experiments were operated in accordance with the institutional guidelines. GICs of low passage (<20) were used for transplantation at the density of 1 × 10^3^ spheres per 10 µL in phosphate buffered saline (PBS). The suspension of GICs was gently injected into the right corpus striatum of the four‐ to six‐week‐old female NOD/SCID mice (Shanghai SiLaiKe Laboratory Animal Co. Ltd). The animals were kept in individually pathogen‐free ventilated cages with controlled temperature and humidity.

### Preparation and Characterization of the Nanoparticles

The DOPA‐coated CaP cores were prepared through the water‐in‐oil reverse microemulsion method. First, 50 µL of 2 mg mL^−1^ microRNA and 300 µL of 2.5 m CaCl_2_ were dispersed into a cyclohexane/Igepal CO‐520 (71/29, V/V) solution (20 mL) to form the calcium phase. In the meanwhile, 300 µL of 12.5 × 10^−3^ m Na_2_HPO_4_ (pH > 9) was added dropwise to another cyclohexane/Igepal CO‐520 (71/29, V/V) solution (20 mL) to form the phosphate phase. One‐hundred and twenty microliters of DOPA (20 × 10^−3^ m) in chloroform was then added to the phosphate phase. The two phases were mixed by stirring for 45 min to form microemulsion. After that, 40 mL of ethanol was added. The precipitates were collected through centrifugation at 12 500 g for 20 min. The obtained miRNA‐loaded CaP cores were dissolved in 3 mL of chloroform and stored at −20 °C for further modification. To prepare miR34a‐LNC, the miR34a‐loaded CaP cores were mixed with 4 mg DMPC and evaporated on Büchi Rotavapor R‐200 (Büchi, Germany) at 40 °C for 1 h to remove chloroform under vacuum. The obtained lipid film was rehydrated with 4 mL of 0.01 m PBS buffer (pH 7.4). The liposome solution was named as miR34a‐LNC. To prepare miR34a‐LNPs, miR34a‐LNC was incubated with ApoE3 at the ratio of 1:8 (ApoE3: DMPC, w/w) at 37 °C for 36 h. To prepare miR34a‐SLNPs, miR34a‐LNC was first incubated with the SDF1 mimic peptides (FH27, FH29 and FH38 peptides) at the ratio of 1:100 (peptides: DMPC, molar ratio) for 24 h and then incubated with ApoE3 at the ratio of 1:8 (ApoE3: DMPC, w/w) for another 24 h. The morphology and size of NC‐SLNPs were detected under a JEM‐1400 plus TEM (JEOL, Japan) after negative staining with 1.75% sodium phosphotungstate solution. Zeta potential and particle size distribution of the nanoparticles were determined with a dynamic light scattering detector (Zetasizer Nano‐ZS90, Malvern Instruments, UK). To evaluate the stability of nanoparticles in serum, naked NC‐miRNA, NC‐LNPs, and NC‐SLNPs (FH27‐NC‐SLNPs, FH29‐NC‐SLNPs, and FH38‐NC‐SLNPs) were incubated with 50% fetal bovine serum (FBS) at 37 °C for 0, 2, 4, and 8 h. The samples were then subjected to electrophoresis with 2% agarose gel at a constant voltage of 90 V for 30 min and the image was captured by an ODYSSEY infrared imaging system.

### Qualitative and Quantitative Analysis of the Cellular Uptake of SLNPs

GICs were seeded at the density of 200 spheres per well into 24‐well plates, and cultured overnight. Then the cells were incubated with the three DiI‐SLNPs at 37 °C for 3.5 h at the DMPC concentration of 20 µg mL^−1^. After that, the cells were washed with PBS buffer, and fixed with 4% formaldehyde for 20 min. Then the spheres were gently pipetted into single cells and subjected to flow cytometry analysis. For a qualitative experiment, the cell spheres were stained with CXCR4 antibody to analyze the colocalization of DiI‐SLNPs and CXCR4. To determine whether the uptake of SLNPs was CXCR4‐stimulated macropinocytosis dependent, GICs were preincubated with 150 × 10^−6^ m EIPA, an endocytosis inhibitor of macropinocytosis, for 1 h. After that, the three DiI‐SLNPs were added into each well at the DMPC concentration of 20 µg mL^−1^ and cotreated with 1 mg mL^−1^ FITC‐Dextran (70 kD) for another 1.5 h. The cells were then fixed and qualitatively analyzed by confocal microscopy. Furthermore, to test the CXCR4 dependence, GICs were transfected with a lentivirus‐mediated gene transfer system containing CXCR4 shRNA (HanYin Biotech, Shanghai, China) and selected with 2 mg mL^−1^ puromycin for 3 d. The shRNA sequence of CXCR4 shRNA is GATCCGTGCCGTGGCAAACTGGTACTTCAAGAGAGTACCAGTTTGCC ACGGCATTTTTTG. Efficient knockdown of CXCR4 was confirmed by Western blotting. The cellar uptake of SLNPs in these CXCR4 knocked down cells was qualitatively analyzed through confocal microscopy and quantitatively analyzed through flow cytometry.

### In Vivo Enhanced GICs Targeting Efficiency

Eighteen NOD/SCID mice bearing GICs‐derived glioma were divided into six groups in random and intravenously given with Cy5‐LNPs and Cy5‐SLNPs at the DMPC concentration of 20 mg kg^−1^. At 4 h after administration, the mice were anesthetized and heart perfused with 4% saline and paraformaldehyde sequentially. After that, the fluorescent images of the brains were captured via a Maestro in vivo imaging system. The brains were then collected and imbedded in optimal cutting temperature compound (Sakura, Torrance, CA, USA), frozen at −80 °C and sectioned. The frozen brain sections were stained with SOX2 antibody and observed under the confocal microscope. Another 12 brain tumors from the GICs glioma‐bearing NOD/SCID mice were collected at 4 h postinjection of the Cy5‐labeled nanoparticles at the DMPC concentration of 20 mg kg^−1^. After washing with PBS, the tumors were minced and disaggregated in 0.05% Trypsin‐EDTA and Type 4 Collagenase. Then the tumors cells were stained with CXCR4 antibody and analyzed through flow cytometry.

### Intracellular Fate of miRNA Loaded in SLNPs

To verify that FAM‐NC can escape from lysosomes, LNPs, FH27‐SLNPs, FH29‐SLNPs, and FH38‐SLNPs loaded with FAM‐NC was incubated with GICs for 2, 4, and 12 h. To verify that FAM‐NC can be released from the carrier, GICs were incubated with FAM‐NC and DiI double‐loaded LNPs and SLNPs for 2, 4, and 12 h. The fluorescence signals in the cells were imaged using laser confocal scanning microscopy.

### In vitro Anticancer Activity Analysis

To evaluate the effect of the miR34a‐SLNPs on the self‐renewal ability of GICs, cells were seeded at the density of 2 × 10^4^ cells per well into 24‐well plates and cultured overnight for in vitro colony formation assay. The cells were treated with miR34a‐LNPs and FH38‐miR34a‐SLNPs at the concentration of 5 × 10^−9^, 50 × 10^−9^, and 100 × 10^−9^ m miRNA. Those cells incubated with miRNA‐free cell culture medium served as the negative control. Forty‐eight hours later, the culture medium was replaced with fresh high glucose DMEM containing 10 % FBS. The cells were allowed to grow for another 72 h. After that, the cells were washed with PBS and fixed in 4% formaldehyde for 20 min. Then the cell clones were stained with 0.5% crystal violet solution for 3 min and washed until the background is clear.

### Survival Analysis

In vivo anticancer activity of the GICs‐targeted formulation was evaluated in mice bearing patient‐derived GICs. The treatment was performed at 7, 10, 13, 16, and 19 d after the inoculation. The GICs‐bearing mice were divided into six groups randomly, and treated with saline, TMZ, MiR34a‐LNPs, FH38‐MiR34a‐SLNPs, TMZ+MiR34a‐LNPs, and TMZ+FH38‐MiR34a‐SLNPs, respectively. The nanoparticles were given intravenously at the miR34a dose of 0.36 mg kg^−1^ and TMZ was given via oral gavage at the concentration of 100 mg m^−2^. The survival of each group was recorded and analyzed.

### Biosafety Evaluation

To evaluate the safety of miR34a‐SLNPs, healthy Institute of Cancer Research (ICR) mice (4–6 weeks) were divided into three groups randomly and intravenously injected with saline, miR34a‐LNPs and FH38‐miR34a‐SLNPs, respectively, for totally five administrations every third day. Twenty‐four hours after the fifth injection, the blood samples were collected for hematological and blood biochemical determinations before the mice were sacrificed. After euthanasia, the major organs such as brain, heart, lung, liver, spleen, and kidney were collected and fixed in 4% formaldehyde for further histological analysis.

### CXCR4 Expression Data Obtained from Ivy Glioma Atlas Project

Data about gene expression on primary GBMs were obtained from Ivy Glioma Atlas Project (Ivy Glioma Atlas Project. Available from: glioma.alleninstitute.org). Z‐score normalized expression of CXCR4 was downloaded from the RNA‐Sequencing data set (Available from: glioma.alleninstitute.org/rnaseq/search/index.html).

### Statistical Analysis

The data were presented as mean ± s.d. Unpaired student's *t*‐test (two‐tailed) was used for two‐group comparison. One‐way ANOVA (analysis of variance) with Bonferroni tests was applied for multiple‐group analysis. Statistical significance was defined as *P* < 0.05.

## Conflict of Interest

The authors declare no conflict of interest.

## Supporting information

Supporting Information

Supplemental Movie 1
