# Supplementary material for: Tailored Lipoprotein‐Like miRNA Delivery Nanostructure Suppresses Glioma Stemness and Drug Resistance through Receptor‐Stimulated Macropinocytosis
Source: Adv Sci (Weinh). 2020 Jan 20;7(5):1903290. doi: 10.1002/advs.201903290 (PMC7055550; doi:10.1002/advs.201903290)
Supplement: Supplementary file 1 — Supporting Information [file ADVS-7-1903290-s001.pdf]

Copyright WILEY-VCH Verlag GmbH & Co. KGaA, 69469 Weinheim, Germany, 2020.

ADVANCED SCIENCE
Open Access

Supporting Information

for *Adv. Sci.,* DOI: 10.1002/advs.201903290

Tailored Lipoprotein-Like miRNA Delivery Nanostructure Suppresses Glioma Stemness and Drug Resistance through Receptor-Stimulated Macropinocytosis

Gan Jiang, Huan Chen, Jialin Huang, Qingxiang Song, Yaoxing Chen, Xiao Gu, Zhenhuan Jiang, Yukun Huang, Yingying Lin, Junfeng Feng, Jiyao Jiang, Yinghui Bao, Gang Zheng, Jun Chen, Hongzhuan Chen,* and Xiaoling Gao**

Copyright WILEY-VCH Verlag GmbH & Co. KGaA, 69469 Weinheim, Germany, 2020.

Supporting Information

Tailored Lipoprotein-Like miRNA Delivery Nanostructure Suppresses Glioma Stemness and Drug Resistance via Receptor-Stimulated Macropinocytosis

Gan Jiang, Huan Chen, Jialin Huang, Qingxiang Song, Yaoxing Chen, Xiao Gu, Zhenhuan Jiang, Yukun Huang, Yingying Lin, Junfeng Feng, Jiyao Jiang, Yinghui Bao, Gang Zheng, Jun Chen*, Hong-Zhuan Chen*, Xiao-Ling Gao*

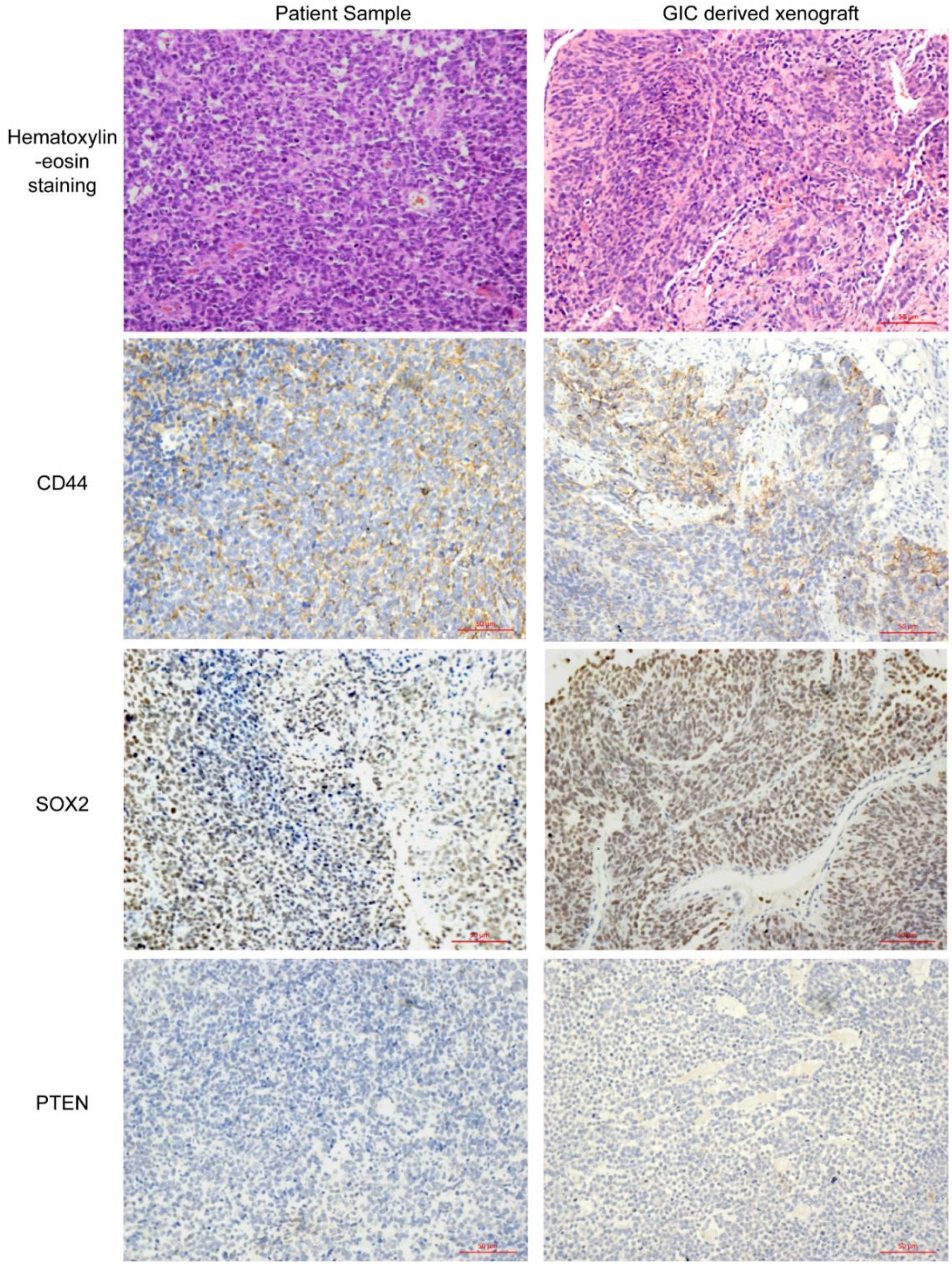

Figure S1. Validation of the phenotype of Glioma initiating cells derived xenograft compared with the patient sample. The patient tumor sample and GICs derived xenograft were stained with H&E, CD44, SOX2 and PTEN antibodies. The patient tumor sample and GICs derived xenograft sample both showed the strongly positive expression of CD44 and SOX2 and negative expression of PTEN. Scale bar, 50 μm.

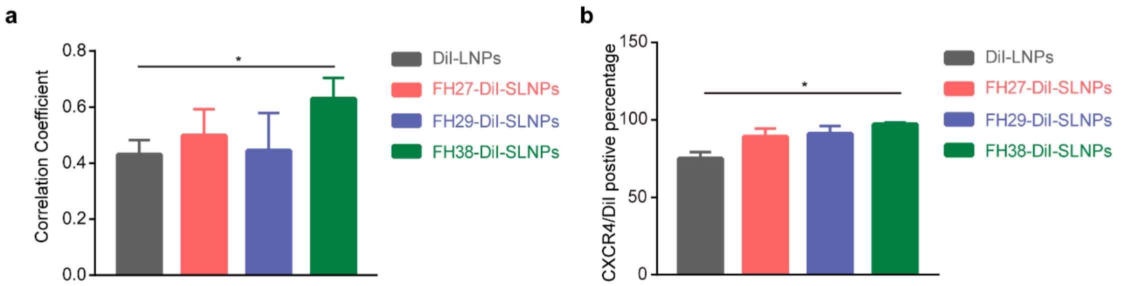

Figure S2. Quantitative analysis of the colocalization of DiI-SLNPs to CXCR4. a) Pearson's correlation coefficient of DiI-SLNPs co-localization to CXCR4. b) The co-localization percentage of DiI-SLNPs to CXCR4. Data represent mean±s.d. (n=3). The significance of the differences was evaluated by one-way ANOVA followed by Bonferroni test. (**p*< 0.05)

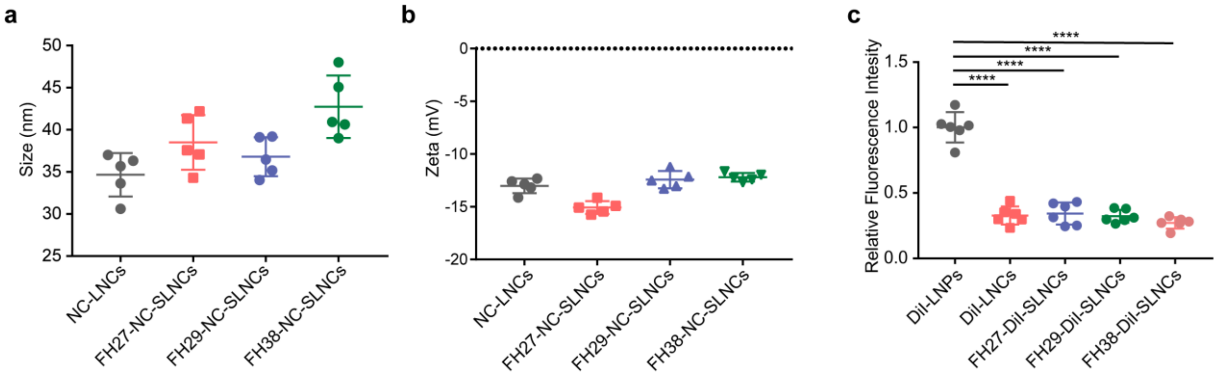

Figure S3. Incorporation of ApoE3 affects the features of LNC and facilitates cellular uptake of the nanoparticles. a) Particle size distribution of NC-SLNCs (FH27/FH29/FH38) under dynamic light scattering. b) Particle zeta potential of NC-SLNCs (FH27/FH29/FH38). c) Without the incorporation of ApoE3 led to relatively low cellular uptake of DiI-LNCs and DiI-SLNCs (n=6). The significance of the differences was evaluated by one-way ANOVA followed by Bonferroni test. (****p< 0.0001)

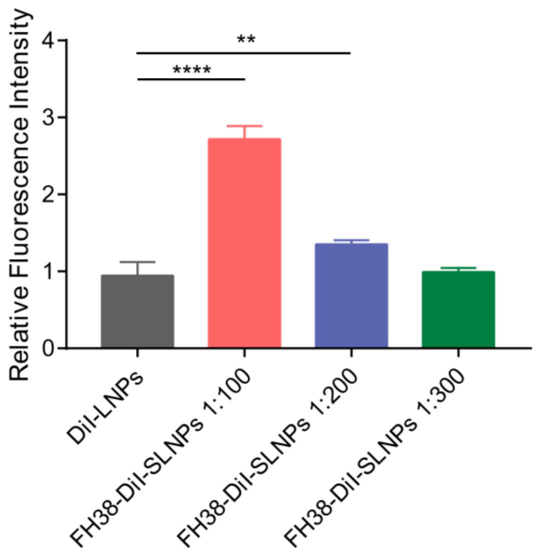

Figure S4. Different peptide loading ratios (peptide:DMPC 1:100, 1:200 and 1:300, molar ratio) led to different cellular uptake of FH38–SLNPs in GICs (n=3). The higher ratio of FH38 peptide led to the more efficient internalization of FH38-SLNPs. The significance of the differences was evaluated by one-way ANOVA followed by Bonferroni test. (**$p < 0.01$, ****$p < 0.0001$)

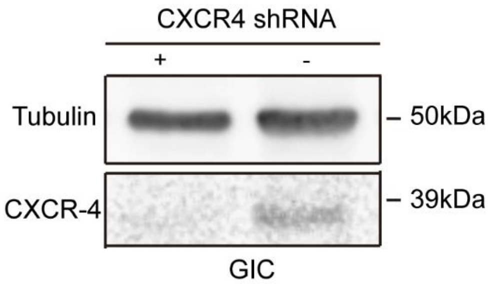

Figure S5. Western blot analysis of CXCR4 expression following gene knockdown. Western Blot analysis confirmed the lower CXCR4 protein level in GICs after knocking down via an shRNA expression lentivirus system.

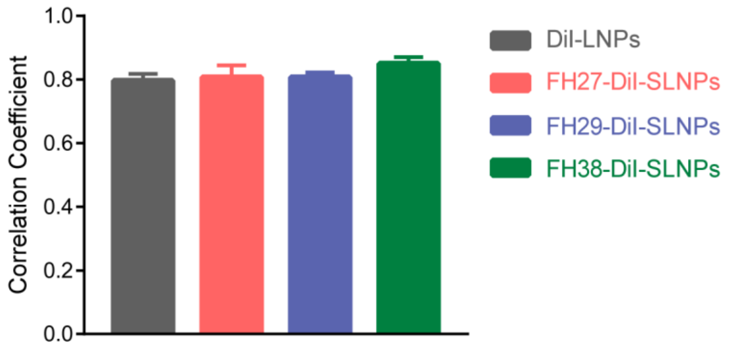

Figure S6. Quantitative analysis of the colocalization between DiI-SLNPs and FITC-dextran. Pearson's correlation coefficient of DiI-SLNPs co-localization to FITC-dextran in GICs model. Data represent mean±s.d. (n=3). The significance of the differences was evaluated by one-way ANOVA followed by Bonferroni test.

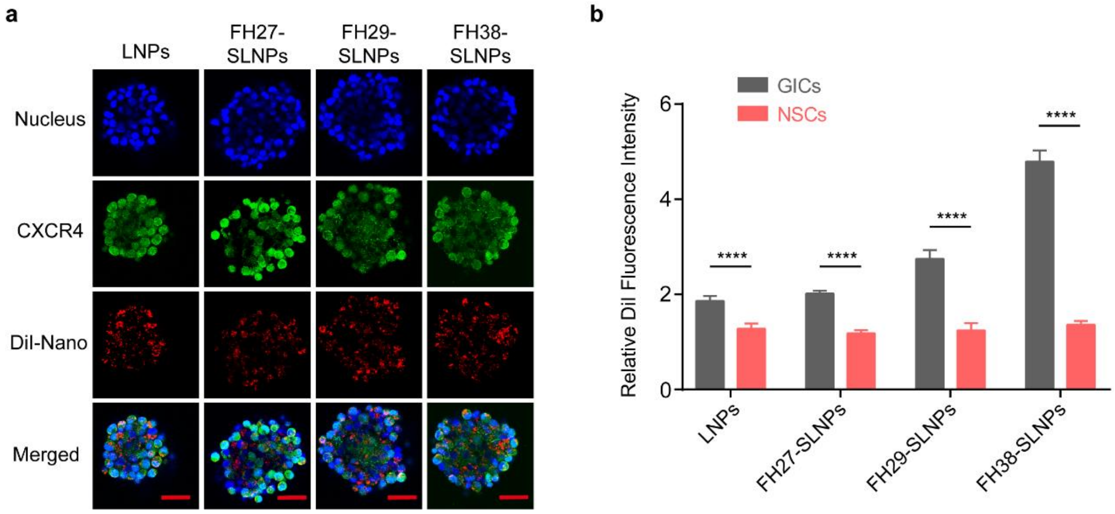

Figure S7. Cellular uptake of DiI-LNPs, FH27-SLNPs, FH29-SLNPs and FH38-SLNPs in NSCs were lower than that in GICs as evidenced by confocal analysis. a) Cellular uptake of DiI-SLNPs in NSCs derived from mice. Scale bar, 50 µm. b) Semi-quantified analysis of the cellular uptake of DiI-LNPs, FH27-SLNPs, FH29-SLNPs and FH38-SLNPs in NSCs and GICs. The significance of differences between the GICs and NSCs groups (****$p < 0.0001$) was evaluated by two-tailed Student's *t*-test.

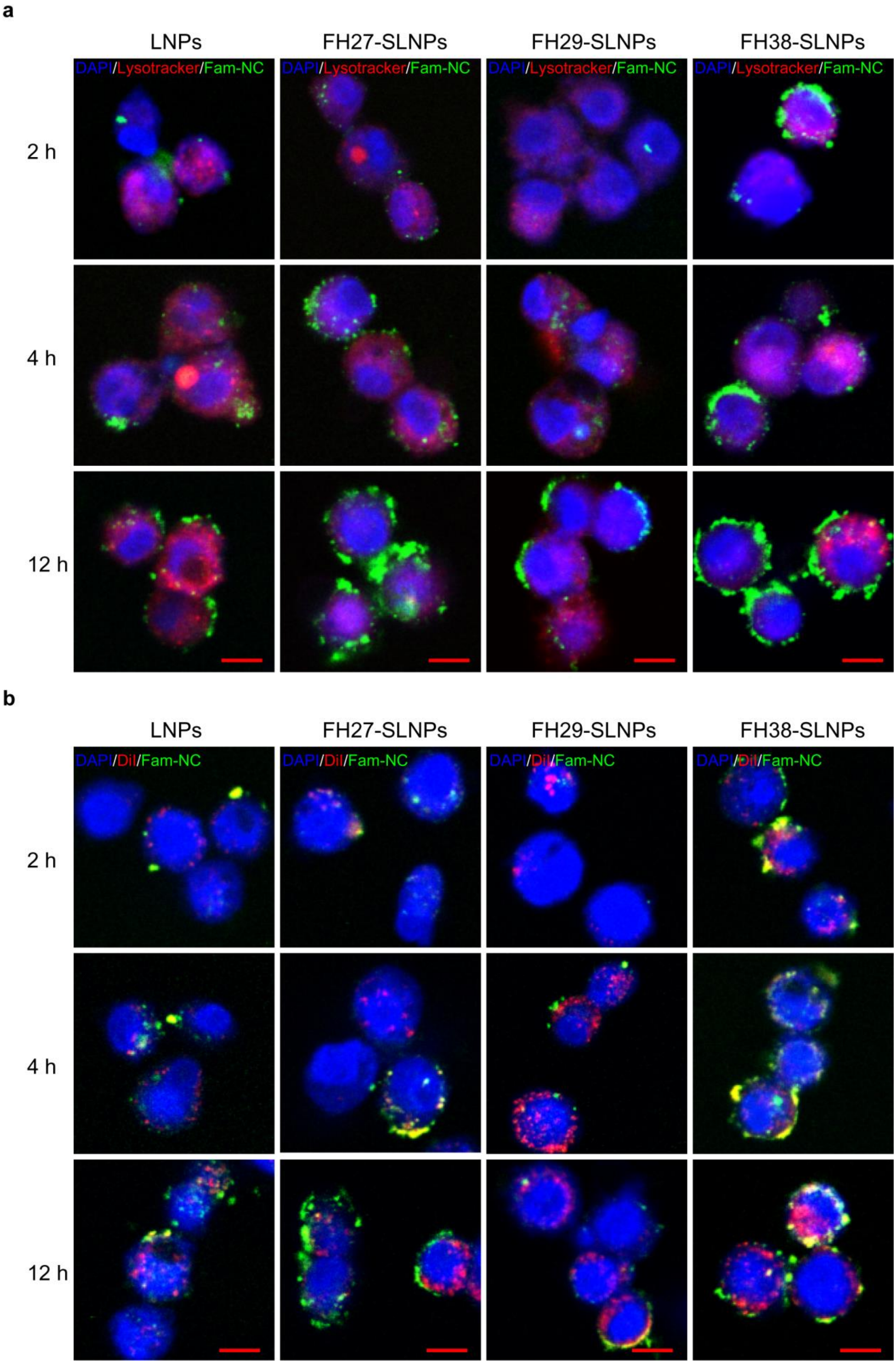
a
LNPs
FH27-SLNPs
FH29-SLNPs
FH38-SLNPs
DAPI/Lysotracker/Fam-NC
2 h
4 h
12 h
b
DAPI/DiI/Fam-NC

Figure S8. SLNPs mediated efficient intracellular delivery of miRNA in GICs. a) Lysosome escape of FAM-NC (green) loaded in SLNPs after incubation for 2, 4 and 12 h. Lysosome was indicated by LysoTracker Red. Nucleus was stained by DAPI (blue). (b) Dissociation of FAM-NC (green) from its carrier after incubation for 2, 4 and 12 h. DiI (red) was inserted as the fluorescent probe of LNPs and SLNPs. Nucleus was stained by DAPI (Blue). Scale bar: 10 μm.

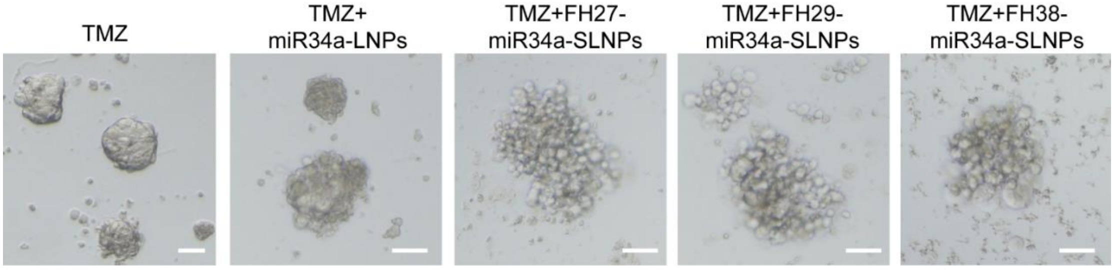

Figure S9. The morphology of GICs following the treatment with miR34a-SLNPs and TMZ. Bright field images of GICs after treatment with 50 μM TMZ and TMZ with miR34a-LNPs and miR34a-SLNPs at the concentration of 50 nM miR34a for 24 h. Scale bar,100 μm.

Movie S1. Patient-derived glioma-initiating cells internalized DiI-LNPs and DiI-FH38-SLNPs as demonstrated through a live-cell imaging system. The GICs were cultured under the standard condition. DiI-LNPs and FH38-DiI-SLNPs were directly added into the dish at the DMPC concentration of 20 μg/ml. GICs captured more FH38-DiI-SLNPs than DiI-LNPs.
